# 'Physical activity at home (PAAH)', evaluation of a group versus home based physical activity program in community dwelling middle aged adults: rationale and study design

**DOI:** 10.1186/1471-2458-11-883

**Published:** 2011-11-24

**Authors:** Nicole Freene, Gordon Waddington, Wendy Chesworth, Rachel Davey, John Goss

**Affiliations:** 1 Faculty of Health, University of Canberra, Canberra, Australia; 2 Physiotherapy, Faculty of Health, University of Canberra, Canberra, Australia; 3 Centre for Research & Action in Public Health, Faculty of Health, University of Canberra, Canberra, Australia

## Abstract

**Background:**

It is well recognised that the adoption and longer term adherence to physical activity by adults to reduce the risk of chronic disease is a challenge. Interventions, such as group and home based physical activity programs, have been widely reported upon. However few studies have directly compared these interventions over the longer term to determine their adherence and effectiveness. Participant preference for home based or group interventions is important. Some evidence suggests that home based physical activity programs are preferred by middle aged adults and provide better long term physical activity adherence. Physiotherapists may also be useful in increasing physical activity adherence, with limited research on their impact.

**Methods:**

'Physical Activity at Home' is a 2 year pragmatic randomised control trial, with a non-randomised comparison to group exercise. Middle-aged adults not interested in, or unable to attend, a group exercise program will be targeted. Sedentary community dwelling 50-65 year olds with no serious medical conditions or functional impairments will be recruited via two mail outs using the Australian federal electoral roll. The first mail out will invite participants to a 6 month community group exercise program. The second mail out will be sent to those not interested in the group exercise program inviting them to take part in a home based intervention. Eligible home based participants will be randomised into a 6 month physiotherapy-led home based physical activity program or usual care. Outcome measures will be taken at baseline, 6, 12, 18 and 24 months. The primary outcome is physical activity adherence via exercise diaries. Secondary outcomes include the Active Australia Survey, accelerometry, aerobic capacity (step test), quality of life (SF-12v2), blood pressure, waist circumference, waist-to-hip ratio and body mass index. Costs will be recorded prospectively and qualitative data will be collected.

**Discussion:**

The planned 18 month follow-up post intervention will provide an indication of the effectiveness of the group and home based interventions in terms of adherence to physical activity, health benefits and cost. If the physiotherapy-led home based physical activity program is successful it could provide an alternative option for physical activity program delivery across a number of settings.

**Trial registration:**

Australia and New Zealand Clinical Trials Register (ANZCTR): ACTRN12611000890932

## Background

The incidence of chronic disease dramatically increases in middle age. Conditions such as cardiovascular disease, type II diabetes and cancer are mostly experienced by people in the middle or older age groups [1]. A major risk factor for chronic disease is physical inactivity [2]. By improving physical activity levels towards the recommended level in this age group, it can have a significant effect on their quality of life and future health [3,4].

In 2004-5 the Australian Bureau of Statistics reported approximately 70% of Australians aged 15 years or more were classified as sedentary or having low levels of physical activity [5]. To increase our physical activity levels a number of health promotion strategies have been employed, such as General Practitioner advice, mass media campaigns, tailored information for communities and community programs targeting groups or individuals [6]. Yet physical activity interventions in middle and older age adults have reported difficulties with the adoption and adherence to physical activity with a number of barriers identified [7,8].

Physiotherapy-led home based programs may be an alternative option to increase the adoption and long term adherence to physical activity. As primary health care providers physiotherapists are well placed to promote physical activity in both the private and public sector for primary prevention of chronic disease [9]. Physiotherapists prescribe exercise for a wide range of conditions and comorbidities utilising a strong background in disease pathologies and body systems. They are well equipped to provide a thorough assessment, individually tailored prescription of exercise and appropriate counselling to achieve an increase in physical activity [10,11]. However, primary prevention of chronic disease via physical activity programs appears to be under utilised in physiotherapy [12]. Shirley [9] found that physiotherapists were far more likely to incorporate physical activity advice into a regular treatment session for a condition rather than a non-treatment one-on-one or group physical activity consultation. Further investigation of physical activity promotion and physiotherapy is needed to determine its impact.

One of the barriers to physical activity adoption may be that individuals prefer to exercise on their own. There is some evidence that a large number of community dwelling individuals may not be interested in group exercise but would undertake exercise on their own in their local area if they had access to appropriate exercise information and or supports. Wilcox reports from a large community survey conducted in the USA that 69% of middle-aged and 67% of older adults preferred to exercise on their own with some instruction rather than in a group exercise class [13]. Yardley reports that 41% of respondents in a large survey conducted in the UK over the age of 54 would not attend group sessions for falls prevention [14]. Some people may also not be suited to groups, particularly those that are lonely and depressed and have decreased cognition [8].

Community based physical activity studies have found that longer term retention of participants within an intervention is difficult [15,16]. Jancey [8] in a 6-month community physical activity intervention for older adults found that participants lost to attrition (35%) came from areas of lower socioeconomic status, were overweight and less physically active, had lower walking self-efficacy scores and higher loneliness scores. Participants reported that they were unable to continue with the physical activity program due to work and travel commitments, and group times being unsuitable. Attrition was not related to age, relationship status, level of education, or gender.

Participant choice and suitability of physical activity programs needs to be considered in order to increase the adoption and adherence to physical activity. A number of physical activity promotion strategies are group or centre based. Home based physical activity programs have been investigated although many appear not to be completely home based, involving some group component or attendance at a centre for assessments [17,18]. To our knowledge there are no known trials reported in the literature of targeted interventions for those individuals who do not wish to take part in group programs.

There is also some evidence that home based programs provide better long term adherence to exercise than centre based programs [19]. This Cochrane review included only 6 studies on home versus centre based physical activity programs in adults older than 50 years. The largest and highest quality rating study in this review found that there was a significantly higher adherence rate to physical activity in the home based program compared with the centre based program, especially in the long term [20]. King [20] reported that at 12 months following the initiation of group and home based interventions, the group intervention had an approximately 50% adherence to exercise, while the home based intervention had a 70% adherence to exercise. Recommendations from this review included investigation of reasons for better adherence in home based programs and exploring the cost-effectiveness of centre and home based programs.

Individual home based physical activity programs with telephone support can be a viable strategy for home based physical activity program provision [18,21]. Telephone counselling has been found to be effective in promoting physical activity in middle-aged and older adults in both healthy and chronic illness samples and has the potential for being a lower cost and more convenient alternative to face-to-face contact [21,22].

Therefore by targeting middle aged adults, where the number of chronic diseases begins to increase noticeably, with a physiotherapy-led home based physical activity program it may increase the adoption and adherence to physical activity over the longer term. By comparing the home based intervention with telephone support to a standard community group exercise program and usual care we will determine the health benefits, longer term adherence to physical activity and the cost effectiveness of delivering such programs.

We hypothesize that:

1. There are a large number of individuals in the community aged 50-65 years who are not interested in group exercise, a commonly used method to increase the population's level of physical activity in the community.

2. A proportion of this group would commence exercising if an alternative method of increasing physical activity was available, such as a home based approach.

3. The home based physical activity program would produce health benefits equivalent to those seen in the group based exercise program for sedentary adults 50-65 years old.

4. Those that commence the home based physical activity program are more likely to continue with an increase in physical activity in the long term, as compared to a group based program.

5. A home based approach to increasing physical activity with minimal support is more cost effective than a group based intervention, taking into consideration long term physical activity adherence.

## Methods

### Design

'Physical Activity at Home' is a 2 year, pragmatic, two arm randomised control trial (RCT) targeting middle-aged adults not interested in, or unable to attend, a group exercise program. Participants eligible for the home based intervention will be randomised, via computer generated numbers, to a physiotherapy-led home based physical activity program or usual care.

Comparison of the physiotherapy-led home based physical activity program and usual care to a non-randomised group exercise program will also be completed. The group exercise program has been included to replicate a standard community physical activity program for middle-aged adults. Using a quasi-experimental design the three interventions will be compared to determine the long term adherence to physical activity, health benefits and cost.

### Participants

To be eligible for this study, participants will be between 50 and 65 years old. Participants must be sedentary; that is, no participation in regular moderate or vigorous exercise or physical activity for 30 min 2 or more times a week for at least 6 months. They will have no serious medical conditions that could limit participation in moderate physical activity, such as unstable angina, uncontrolled hypertension, diagnosed or hospitalized with chest pain, heart attack or heart surgery in the past 6 months and no severe functional impairments due to multiple medical or psychiatric diseases. They will not be planning to move from the area within 2 years and only one person per household will be eligible. Participants will be English speaking and have appropriate cognitive skills to provide informed consent and actively engage in the physical activity program. Medical clearance screening will be undertaken using the Sports Medicine Australia (SMA) Pre-Exercise Screening System [23]. If the participant answers 'yes' to any of the SMA screening questions, they will be asked to attend their local medical officer to receive medical clearance before they can be included in the study.

### Recruitment

**Figure 1:**
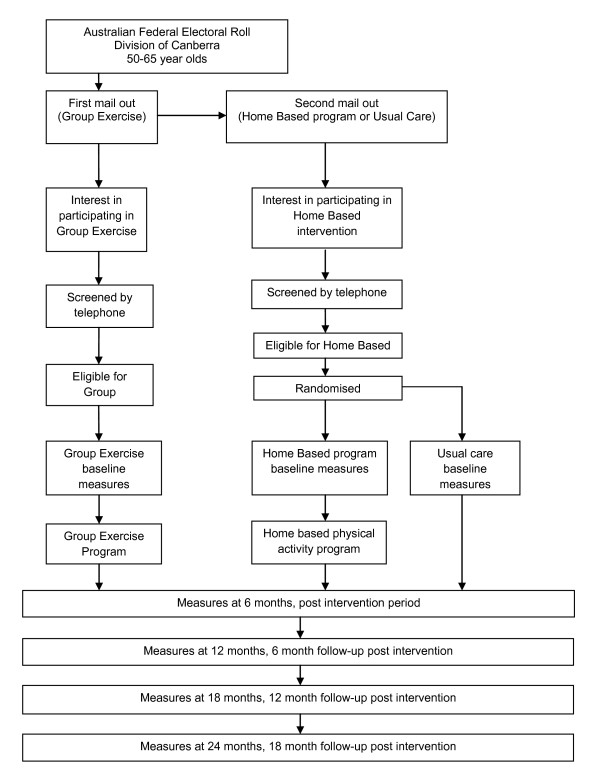
**Flow of participants through the trial**.

Recruitment to the 'Physical Activity at Home' study will take place between February and April 2011. Two mail outs will be conducted using the Australian Electoral Commission (AEC) federal electoral roll to target 50-65 year olds in six suburbs of the Australian Capital Territory (ACT). These suburbs were chosen due to their geographical proximity to a local YMCA. The first letter will ask for expressions of interest in joining a once weekly group exercise program at the local YMCA. The second letter will be sent to those not interested in the group exercise program inviting them to participate in a health project that will consist of either a 6 month physiotherapy-led home based physical activity program or the completion of a number of basic health measures in their homes giving them an indication of their fitness and health status. All individuals that respond to the mail outs will be screened via telephone. The flow of participants through the trial is illustrated in Figure 1.

### Intervention

(i) Home-based intervention

Assessments for the home based program in this study will be conducted in participant's homes eliminating a number of barriers to physical activity adoption as discussed earlier [24]. Participants will be encouraged to invite a support person to attend during their initial assessment. After baseline measures are completed motivational interviewing will be used to devise an individual physical activity program [25]. A physiotherapist will discuss type, frequency, intensity, duration, benefits, barriers, goals, self-monitoring and progression of physical activity, aiming to achieve 30 min of moderate intensity physical activity most days of the week. Participants will be contacted by a physiotherapist via phone providing advice and support 2 weeks after the initial assessment and then monthly over a 6 month period, a total of approximately 6 phone calls.

(ii) Usual Care

Participants randomised to usual care will be visited by the physiotherapist in their homes. Baseline measures for the study will be completed giving participants an indication of their fitness and health status. Participants will receive two brochures 'An active way to better health. National Physical Activity Guidelines for Adults' and 'Dietary Guidelines for Australian Adults' from the Department of Health and Ageing [26,27]. They will not receive any other advice or support for increasing exercise or physical activity levels.

(iii) Group exercise program

Participants in the group based program will attend the YMCA for their baseline measures. Baseline measures will be conducted by the same physiotherapist as the home based interventions. Participants will then have a choice of times for the group based exercise program which will be during business hours replicating similar programs and times offered in this community setting. These sessions will be run by a YMCA fitness instructor at the YMCA once a week, for 60 min, over 6 months. The exercise program will involve upper and lower body strengthening exercises, gross motor skill training and aerobic fitness training. The exercise specifics will be at the discretion of the YMCA fitness instructor, with no involvement from the physiotherapist, aiming to mimic 'usual practice'. Participants will be encouraged to increase their physical activity slowly and gradually in whichever way they prefer outside of the group sessions by the YMCA fitness instructors, aiming to achieve 30 min of moderate intensity physical activity most days of the week. An individual home based exercise program will not specifically be designed for this group.

### Outcome measures

The methods of participant assessment have been carefully considered as assessments are taking place in people's homes. All study measures will be assessed at baseline, 6, 12, 18 and 24 months.

The main outcome measure of this study is long term adherence to physical activity. Adherence will be measured using exercise diaries completed over the 2 year study period, a continuous measure. Exercise diaries have been found to be both reliable and valid [28]. Participants will be encouraged to record date, type, duration and intensity, using the modified Borg rating of perceived exertion scale (RPE) [29], every time they are physically active. Participants will be encouraged to return the diaries in the supplied prepaid envelope at the end of each month over the study period.

Average monthly adherence rates across the 2 year period will be calculated as follows: number of physical activity sessions reported as a percentage of physical activity sessions prescribed for the month. This is similar to the method described by King [20] to determine adherence rates to different physical activity intensities and formats over a 2 year period. The number of sessions prescribed for the month will be based on the World Health Organisation (WHO) physical activity guidelines [3]. That is, 30 min of moderate intensity physical activity, five or more days per week, a total of 20 or more sessions per month. Thirty minutes can be accumulated in 10 min bouts of physical activity per day. Moderate intensity is defined as a rating of 3 or more on the modified Borg RPE scale.

Secondary outcomes include the Active Australia Survey (AAS), accelerometry, aerobic capacity (2 min step test), quality of life (SF-12v2) and disease biomarkers, that is, waist circumference (cm), waist:hip ratio (WHR), blood pressure (mmHg) and body mass index (BMI, kg/m^2^).

The AAS and accelerometry will be used to validate the data collected from the exercise diaries. These two continuous measures will be collected every 6 months over the 2 year study period.

The AAS has been designed to measure participation in leisure time physical activity and to assess the participant's knowledge of current public health messages about the health benefits of physical activity. It offers a short and reliable set of questions and applies to 1 week preceding the interview, including walking for transport. The AAS has been reported as reliable and of acceptable validity [30,31].

Covering the same time period as the AAS, participants will wear an Actigraph GT1M accelerometer [32] for 7 consecutive days. Accelerometers allow an objective measurement of quantity and intensity of movement and have been found to be reliable and valid [33].

The raw data collected by the accelerometer, counts, will be used to obtain the time spent in different physical activity intensities [34]. We will use the Freedson Combination energy expenditure algorithm to determine the intensity cut-points [32]. This outcome variable will be used to investigate whether participants have reached the WHO physical activity guidelines.

Assessment of aerobic capacity is essential to determine whether the completed physical activity has been intense enough to result in an improvement in cardiovascular fitness. The 2 min step test requires little space and equipment, with large studies finding it both reliable and valid [35]. The 2-minute step test protocol involves determining the number of times in 2 min that a person can step in place raising the knees to a height halfway between the patella (kneecap) and iliac crest (front hip bone), a continuous measurement. Only one successful trial will be administered.

The SF-12v2 is a general health status questionnaire which has a 12-item scale producing eight separate sub-scales (physical functioning, physical role functioning, emotional role functioning, social functioning, bodily pain, mental health, vitality and general health perceptions) to assess quality of life [36]. It takes the participant less than 2 min to complete the tool and is a quasi-continuous measurement.

Waist circumference and hip circumference will be measured in centimetres using a tape measure. Blood pressure levels will be obtained using a mercury sphygmomanometer on the right arm of seated subjects. Both measures will be taken twice and then the average will be recorded. BMI (kg/m^2^) will be recorded using a portable set of scales and a stadiometer.

Sociodemographic information will also be collected with questions regarding participant's education level, relationship status, current employment status and the presence of any chronic diseases.

Qualitative data will be collected at the end of the intervention period in both the group exercise and physiotherapy-led home based physical activity programs. Telephone administered semi-structured interviews will be conducted with a sub-sample of both the group and home based interventions. Focus groups will also be conducted, with sub-samples of attendees and non-attendees for both the home based and group interventions. These approaches together with the qualitative data should provide improved data collection, guiding future physical activity programs.

### Sample size

The sample size for this study was based on the study by King [20] cited earlier, reporting a 20% difference in adherence to physical activity between a group and home based intervention at 12 months. Assuming a standard deviation of 35% based on this study and a dropout rate of 25% with a statistical power of 0.8, the sample size needed for each group is 64.

Previous community physical activity intervention studies using various recruitment methods, including utilisation of the AEC federal electoral roll, report a recruitment rate of 10-20% [20,24]. Therefore it was estimated that approximately 2000 letters need to be sent in the first mail out to recruit a suitable sample size.

### Statistical methods

Data will be analyzed according to group assignments, regardless of how many participants actually complete the study, the intent-to-treat principle. A maximum of four attempts will be made to contact participants that did not comply with the intervention protocols so outcome measures can be obtained, making the analysis more complete [37].

An intention-to-treat analysis may result in an underestimate of treatment effect, making it harder to find a significant difference [37]. Therefore data will be analyzed using both intention-to-treat and on-protocol analyses to determine if the two methods yield the same or different results. This justifies the allowance of a 25% drop-out rate.

Significance level will be set at *p *< 0.05. All analyses will be conducted using SPSS version 18.0.

### Randomised control trial

The primary analysis for the RCT will use repeated measures analysis of covariance (ANCOVA). All analyses will be adjusted for baseline values. Additional covariates to be considered include: gender, age and employment status, an ordinal measurement.

### Other analyses

A subsidiary comparison with the group exercise program will use the ANCOVA as described above, with the addition of the third intervention group.

### Cost-effectiveness analysis

Cost components for recruitment of participants and cost of the interventions will be recorded prospectively during the 2 year study period. Cost items include participant travel costs and resource use, for example, hospitalisations and GP visits, which will be collected via the 6-monthly questionnaires over the 2 year period. This data will be used in conjunction with an estimate of the health benefits, measured in terms of Disability Adjusted Life Years (DALYs), to undertake a cost-effectiveness analysis comparing the two arms of the study. Disability adjusted life years will be estimated using the method developed by Murray [38]. Health status will be measured using preference-based health status scores derived from individual SF-12 questionnaire responses [39]. The cost-effectiveness analysis will be conducted prospectively alongside the trial to compare costs per DALY change in the trial arms. Cost-effectiveness will be calculated as the ratio of the difference in costs between the home based physical activity program and usual care divided by the difference in DALYs between the two groups. The non-parametric bootstrap method (using 1000 replications) will be used to derive confidence intervals for the incremental cost-effectiveness ratio (ICER) over the follow-up period [40]. Cost-effectiveness acceptability curves [41] will be constructed from these data to provide estimates of the probability that, for a given level of the cost per DALY--the so-called 'ceiling' level, the home based physical activity program is more cost-effective than usual care.

Similar analysis will be conducted to compare cost-effectiveness between the home based interventions and the group exercise program.

## Discussion

Over a 2 year period the 'Physical Activity at Home' study will test the effectiveness, in terms of long term physical activity adherence, health benefits and cost, of a physiotherapy-led home based physical activity program. Specifically it will target middle aged adults who are less likely to adopt and maintain physical activity, as they do not access the currently available community group exercise programs. With few studies reporting on the long term adherence to physical activity post intervention and no known studies reporting on targeted physical activity interventions for those not interested in group or centre based programs, if this program is successful it could allow more individuals to increase their physical activity levels towards that needed to achieve the associated health benefits.

One of the strengths of this trial is that the inclusion criteria are broad proposing that there is some benefit from physical activity for almost everyone [42]. The recruitment method also attempts to minimize self-selection and recruitment of highly motivated volunteers by using the electoral roll, allowing a more representative sample [24]. Phone coverage is widespread in Australia therefore recruitment and providing the home based intervention via this method should also limit selection bias. All measurements will be performed by the same person and the sample size to be recruited should provide adequate statistical power to detect a significant difference in the main outcome measure.

A limitation of this study is the lack of randomisation to the group and home based interventions limiting the ability to attribute outcomes to treatment for all three groups. There is also a lack of blinding, as the principal researcher will be conducting all assessments and providing the home based intervention. Another possible limitation is the type of personnel conducting the interventions is not standardised, that is, a physiotherapist as compared to a fitness instructor.

If successful, this physiotherapy-led home based physical activity program should improve physical activity levels over the longer term, possibly in a cost-effective manner and particularly for those individuals who are not interested in, or unable to access, group exercise programs. This program could provide an alternative option for physical activity program delivery to sections of the population with various risk factors and/or diseases, a wider age range and across a number of different settings, such as rural and remote.

## Ethical approval

This trial was approved by the University of Canberra Committee for Ethics in Human Research in November 2009 (Project number 09-97).

## Competing interests

The authors declare that they have no competing interests.

## Authors' contributions

All authors contributed to the study design and development of the trial protocol. NF is the principal investigator and trial manager of the Physical Activity at Home study. NF drafted the paper and GW, WC, RD and JG contributed to subsequent drafts. All authors read and approved the final manuscript.

## Pre-publication history

The pre-publication history for this paper can be accessed here:

http://www.biomedcentral.com/1471-2458/11/883/prepub
